# Metabolic maturity patterns in neonates: dissecting the interactive effects of gestational age and birth weight on metabolic profiles

**DOI:** 10.3389/fped.2026.1800632

**Published:** 2026-05-01

**Authors:** Hua Tang, Yanqiong Ma, Ying Zhou, Huiming Yan, Huiping Zhang

**Affiliations:** 1 Department of Medical Genetics, Hunan Province Maternal and Child Health Hospital, Changsha, China; 2 NHC Key Laboratory of Metabolic Cardiovascular Diseases Research, Ningxia Medical University, Yinchuan, China

**Keywords:** interaction effects, metabolic maturation, newborn screening, reference intervals, tandem mass spectrometry

## Abstract

**Background:**

Newborn screening (NBS) based on tandem mass spectrometry (MS/MS) is essential for the early identification of inherited metabolic disorders. However, physiological immaturity in preterm and low-birth-weight (LBW) infants frequently alters metabolite concentrations, thereby increasing the risk of false-positive results.

**Objective:**

This study aimed to clarify the independent and interactive effects of gestational age (GA) and birth weight (BW) on neonatal amino acid and acylcarnitine profiles, with the goal of improving screening precision in vulnerable populations.

**Methods:**

We conducted a retrospective analysis of 147,643 neonates screened in Hunan Province, China. The cohort was stratified into seven GA groups and five BW groups. One-way and two-way analyses of variance (ANOVA), together with principal component analysis (PCA), were used to characterize main effects, interaction effects, and global metabolic clustering.

**Results:**

Metabolic profiles followed distinct developmental trajectories. Preterm infants (<37 weeks) showed significantly elevated amino acid concentrations (e.g., tyrosine and arginine) but relative attenuation of long-chain acylcarnitine levels, suggesting an altered balance between protein turnover and fatty acid oxidation. Importantly, significant GA × BW interactions were detected for multiple markers, indicating that the metabolic impact of birth weight varied according to gestational maturity.

**Conclusion:**

Neonatal metabolic maturity is shaped by the synergistic interplay between intrauterine duration and fetal growth. The characteristic “high amino acid/low long-chain acylcarnitine” pattern observed in preterm infants, especially in those with lower birth weight, supports the need for individualized reference intervals in newborn screening.

## 1 Introduction

Newborn screening (NBS) is among the most successful public health interventions in modern preventive medicine and has fundamentally improved the early detection of inborn errors of metabolism (IEMs) (1). The introduction of tandem mass spectrometry (MS/MS) has greatly expanded screening capacity by enabling simultaneous measurement of amino acids and acylcarnitines from dried blood spots (2, 3).

Nevertheless, diagnostic precision remains suboptimal in premature and low-birth-weight (LBW) infants, a population characterized by physiological immaturity, altered nutrient handling, and rapidly changing metabolic homeostasis (4–7). Compared with term neonates, preterm infants often have immature hepatic enzyme activity, incomplete mitochondrial fatty acid oxidation capacity, and distinct nutritional exposures, all of which may substantially influence screening biomarkers independent of disease status.

Although interest in gestationally tailored interpretation has increased, the literature on metabolic reference intervals in preterm infants remains incomplete (8). Several recent studies have confirmed that gestational age (GA) and birth weight (BW) influence neonatal metabolite concentrations (9–11). However, most prior analyses evaluated these factors separately, whereas in clinical practice GA and BW are biologically intertwined. This distinction matters because the metabolic effect of low birth weight may differ markedly between a preterm infant and a term infant who is small for gestational age. A more clinically useful framework therefore requires explicit assessment of their interaction.

## 2 Materials and methods

### 2.1 Study population and ethics

This retrospective study used data obtained from the Hunan Provincial Newborn Screening Center. We reviewed the records of all neonates who underwent expanded newborn screening by tandem mass spectrometry between January 1, 2023, and December 31, 2023. After applying the predefined inclusion and exclusion criteria, 147,643 neonates were enrolled in the final analysis.

Inclusion criteria were as follows: (1) neonates who underwent routine expanded newborn screening during the study period; (2) availability of complete demographic and laboratory information, including gestational age, birth weight, sex, age at sampling, and metabolite concentrations; (3) dried blood spot specimens collected at ≥48 h after birth and after adequate feeding; and (4) specimens that met routine analytical and quality-control requirements for MS/MS-based newborn screening (12, 13).

Exclusion criteria were as follows: (1) confirmed inborn errors of metabolism (IEMs); (2) blood transfusion before sample collection; (3) severe congenital malformations, severe infection, or other critical illnesses likely to substantially alter metabolic homeostasis; and (4) missing key clinical variables or poor-quality specimens unsuitable for analysis. These criteria were applied to reduce potential confounding and to ensure that the final cohort was appropriate for evaluating the effects of gestational age and birth weight on metabolic profiles.

The study protocol was reviewed and approved by the Ethics Committee of the Hunan Provincial Maternal and Child Health Care Hospital (Approval No. 2023-S183). Because this was a retrospective study based on de-identified routine screening data, the requirement for informed consent was waived by the ethics committee.

### 2.2 Sample collection and laboratory analysis

Heel-prick blood samples were collected at least 48 h after birth, after confirmation of adequate nutritional intake (either breastfeeding at least six times or documented enteral feeding at least six times), in accordance with national newborn screening practice standards and technical guidelines for tandem mass spectrometry-based newborn screening (12, 13). Dried blood spots were prepared on standardized filter paper cards and transported to the central laboratory under controlled conditions.

Metabolic profiling was performed using tandem mass spectrometry (MS/MS). Sample preparation was carried out with the NeoBase™ Non-derivatized MSMS Kit (PerkinElmer, Turku, Finland) according to the manufacturer's protocol, and analytes were measured on a Xevo TQD tandem mass spectrometer (Waters, Milford, MA, USA). Internal standards and calibration materials supplied with the kit were used throughout the analytical workflow.

A total of 43 metabolic markers were quantified, comprising 11 amino acids (including Alanine, Arginine, Citrulline, etc.), 31 acylcarnitines (ranging from free carnitine C0 to long-chain acylcarnitines), and Succinylacetone.

To ensure analytical accuracy and reproducibility, rigorous quality-control procedures were implemented. Each 96-well plate included two blank controls, two low-level quality-control samples, and two high-level quality-control samples. Internal and external quality-assurance procedures were performed in accordance with the requirements of the National Center for Clinical Laboratories, the provincial newborn screening quality-control program, and CLSI recommendations for newborn screening by tandem mass spectrometry (12).

### 2.3 Grouping strategy

To systematically assess the independent and interactive effects of gestational age (GA) and birth weight (BW) on metabolic profiles, the study population was stratified into predefined subgroups. Based on gestational age, neonates were classified into seven categories: <37 weeks, 37–37 + 6 weeks, 38–38 + 6 weeks, 39–39 + 6 weeks, 40–40 + 6 weeks, 41–41 + 6 weeks, and ≥42 weeks. Based on birth weight, neonates were classified into five categories: <2,500 g, 2,500–2,999 g, 3,000–3,499 g, 3,500–3,999 g, and ≥4,000 g.

#### 2.3.1 Statistical analysis

Data management and statistical analyses were conducted using R software (version 4.4.3; R Foundation for Statistical Computing, Vienna, Austria). Continuous variables are presented as mean ± standard deviation (SD), whereas categorical variables are expressed as counts and percentages. Differences in sex distribution across GA and BW subgroups were evaluated using the Pearson chi-square test.

To evaluate metabolic differences across developmental stages, one-way analysis of variance (ANOVA) was used to compare metabolite concentrations among the seven gestational-age groups and among the five birth-weight groups. *post-hoc* pairwise comparisons were performed when the overall test was significant.

To further dissect the interplay between gestational age and fetal growth, two-way ANOVA models were fitted with GA and BW as fixed factors. These models were used to estimate both the main effects of each factor and their interaction effects on individual metabolites.

Given the large number of simultaneous hypothesis tests, Bonferroni correction was applied to *post-hoc* pairwise comparisons to control the family-wise error rate. Two-sided *P* values < 0.05 were considered statistically significant unless otherwise specified.

Finally, principal component analysis (PCA), an unsupervised multivariate technique, was used to reduce the dimensionality of the high-throughput MS/MS dataset and to visualize global metabolic clustering patterns across developmental subgroups.

## 3 Results

### 3.1 Demographic characteristics

A total of 147,643 neonates who met the inclusion criteria were enrolled in the final analysis. Baseline demographic characteristics stratified by gestational age (GA) and birth weight (BW) are summarized in Table 1.

**Table 1:** Demographic characteristics of the newborn population stratified by gestational age and birth weight (*N* = 147,643).

Variable	Total	Male	Female	*P* value^a^
	No. (%)	No. (%)	No. (%)	
Total population	**147,643 (100.0)**	**78,841 (53.4)**	**68,802 (46.6)**	
**Gestational age (weeks)**				<**0.001**
<37 (Preterm)	6,363 (10.4)	3,677 (57.79)	2,686 (42.21)	
37–37 + 6	15,303 (26.3)	8,636 (56.43)	6,667 (43.57)	
38–38 + 6	38,874 (19.3)	21,288 (54.76)	17,586 (45.24)	
39–39 + 6	48,726 (33.0)	25,123 (51.56)	23,603 (48.44)	
40–40 + 6	31,848 (10.0)	15,562 (48.86)	16,122 (50.62)	
41–41 + 6	6,675 (0.9)	3,126 (46.83)	3,549 (53.17)	
≥42 (Post-term)	44 (0.03)	26 (59.09)	18 (40.91)	
**Birth Weight (g)**				<**0.001**
<2,500 (LBW)	3,842 (2.6)	1,755 (45.68)	2,087 (54.32)	
2,500–2,999	29,477 (20.0)	13,231 (44.89)	16,246 (55.11)	
3,000–3,499	68,829 (46.6)	34,762 (50.50)	34,607 (49.50)	
3,500–3,999	38,125 (25.8)	22,762 (59.70)	15,368 (40.31)	
≥4,000 (Macrosomia)	7,396 (5.0)	4,928 (66.63)	2,468 (33.37)	

LBW, Low Birth Weight.

a *P* values were calculated using the Pearson Chi-square test to compare gender distribution across subgroups.

Bold values indicates statistically significant difference at *P*<0.05.

When categorized by gestational age, the study population was divided into seven subgroups. The largest cohort consisted of infants born at 39–39 + 6 weeks of gestation (*n* = 48,726, 33.0%), whereas preterm infants (<37 weeks) represented 10.4% of the study population. Overall, the cohort was predominantly composed of term neonates.

Regarding gender distribution, there were slightly more males (53.4%) than females (46.6%) in the overall cohort. Statistical analysis revealed significant differences in gender composition across both gestational age (*χ*² test, *P* < 0.001) and birth weight groups (*P* < 0.001). Specifically, the proportion of males was generally higher in the heavier birth weight categories, consistent with known biological growth patterns.

### 3.2 Main effects of gestational age and birth weight on metabolic profiles

One-way ANOVA demonstrated that neonatal metabolic profiles were strongly influenced by developmental maturity. As summarized in Table 2, gestational age significantly affected the concentrations of 40 of the 43 measured biomarkers, indicating a broad maturational effect on both amino acid and acylcarnitine metabolism.

**Table 2:** Concentrations of amino acids and acylcarnitines across different gestational age groups.

Analyte	<37 weeks	37–37 + 6 weeks	38–38 + 6 weeks	39–39 + 6 weeks	40–40 + 6 weeks	41–41 + 6 weeks	≥42 weeks	*F* value^a^	*P* adj^b^
	Mean (SD)	Mean (SD)	Mean (SD)	Mean (SD)	Mean (SD)	Mean (SD)	Mean (SD)		
Amino Acids (*μ*mol/L)									
Alanine (ALA)	304.72 ± 91.69	319.57 ± 90.42	319.77 ± 88.27	325.01 ± 88.71	332.01 ± 90.35	335.69 ± 89.73	362.26 ± 98.41	134.44	<0.001
Arginine (ARG)	10.83 ± 9.44	7.55 ± 6.04	7.51 ± 5.75	7.41 ± 5.67	7.18 ± 5.53	6.78 ± 5.30	9.30 ± 7.04	373.93	<0.001
Citrulline (CIT)	13.66 ± 4.87	13.04 ± 3.52	13.39 ± 3.44	13.73 ± 3.48	13.95 ± 3.57	13.92 ± 3.47	14.60 ± 3.67	157.38	<0.001
Glycine (GLY)	407.86 ± 105.52	450.93 ± 105.52	451.43 ± 98.46	450.67 ± 98.40	454.06 ± 99.84	458.38 ± 103.83	449.82 ± 115.74	206.96	<0.001
Leucine (LEU + ILE + PRO-OH)	136.11 ± 33.86	131.83 ± 31.40	133.64 ± 32.44	135.31 ± 32.32	137.18 ± 32.93	139.38 ± 33.58	143.05 ± 37.87	81.40	<0.001
Methionine (MET)	20.30 ± 5.72	20.00 ± 5.37	20.01 ± 5.20	19.81 ± 5.19	19.78 ± 5.19	19.68 ± 5.01	20.77 ± 5.59	17.07	<0.001
Ornithine (ORN)	79.91 ± 27.44	76.73 ± 22.28	80.49 ± 22.81	82.95 ± 22.55	84.67 ± 22.83	85.55 ± 24.97	86.37 ± 24.38	283.16	<0.001
Phenylalanine (PHE)	44.96 ± 10.75	46.41 ± 10.67	46.05 ± 10.20	46.07 ± 9.92	46.47 ± 11.27	47.10 ± 9.91	45.73 ± 8	30.70	<0.001
Proline (PRO)	151.15 ± 39.58	158.17 ± 39.53	163.98 ± 39.83	167.62 ± 41.50	169.70 ± 41.50	170.93 ± 42.11	175.19 ± 42.13	319.61	<0.001
Tyrosine (TYR)	100.35 ± 49.72	100.38 ± 42.99	95.43 ± 37.95	90.77 ± 34.07	87.01 ± 30.67	85.78 ± 28.90	84.21 ± 33.65	394.98	<0.001
Valine (VAL)	108.60 ± 28.61	110.92 ± 28.14	115.31 ± 28.62	118.73 ± 29.34	121.63 ± 30.17	123.84 ± 31.12	124.88 ± 35.72	434.50	<0.001
Acylcarnitines (μmol/L)									
Free carnitine (C0)	24.47 ± 9.96	21.63 ± 7.96	20.20 ± 7.14	19.93 ± 6.95	19.96 ± 7.10	19.91 ± 7.24	22.17 ± 6.66	465.22	<0.001
Acetylcarnitine (C2)	17.63 ± 8.11	19.47 ± 7.09	18.74 ± 6.43	18.63 ± 6.43	19.10 ± 6.82	20.27 ± 7.22	20.32 ± 7.12	124.02	<0.001
Propionylcarnitine (C3)	1.73 ± 0.90	1.86 ± 0.76	1.73 ± 0.68	1.64 ± 0.63	1.60 ± 0.62	1.58 ± 0.60	1.67 ± 0.56	351.10	<0.001
Isobutyrylcarnitine (C4)	0.25 ± 0.07	0.27 ± 0.07	0.26 ± 0.07	0.25 ± 0.07	0.25 ± 0.07	0.25 ± 0.07	0.25 ± 0.07	157.71	<0.001
Isovalerylcarnitine (C5)	0.15 ± 0.06	0.13 ± 0.05	0.12 ± 0.04	0.12 ± 0.04	0.11 ± 0.05	0.11 ± 0.04	0.13 ± 0.06	817.20	<0.001
Hexanoylcarnitine (C6)	0.069 ± 0.024	0.071 ± 0.025	0.071 ± 0.024	0.070 ± 0.023	0.070 ± 0.025	0.070 ± 0.025	0.072 ± 0.025	10.31	<0.001
Octanoylcarnitine (C8)	0.076 ± 0.026	0.081 ± 0.091	0.079 ± 0.029	0.079 ± 0.027	0.079 ± 0.058	0.079 ± 0.026	0.079 ± 0.026	9.67	<0.001
Decanoylcarnitine (C10)	0.087 ± 0.036	0.098 ± 0.041	0.098 ± 0.039	0.098 ± 0.039	0.098 ± 0.039	0.100 ± 0.040	0.099 ± 0.038	94.00	<0.001
Lauroylcarnitine (C12)	0.089 ± 0.052	0.11 ± 0.057	0.11 ± 0.054	0.11 ± 0.053	0.11 ± 0.053	0.11 ± 0.056	0.12 ± 0.064	181.52	<0.001
Myristoylcarnitine (C14)	0.17 ± 0.085	0.21 ± 0.077	0.20 ± 0.071	0.19 ± 0.068	0.19 ± 0.067	0.20 ± 0.069	0.20 ± 0.085	193.37	<0.001
Palmitoylcarnitine (C16)	2.31 ± 1.37	3.00 ± 1.20	3.04 ± 1.15	3.04 ± 1.15	3.09 ± 1.17	3.24 ± 1.22	3.04 ± 1.39	448.61	<0.001
Stearoylcarnitine (C18)	0.75 ± 0.35	0.90 ± 0.30	0.90 ± 0.29	0.89 ± 0.29	0.89 ± 0.29	0.92 ± 0.30	0.84 ± 0.32	261.22	<0.001
Malonylcarnitine/3-Hydroxybutyrylcarnitine (C3DC + C4OH)	0.15 ± 0.069	0.17 ± 0.072	0.17 ± 0.070	0.17 ± 0.069	0.17 ± 0.071	0.18 ± 0.075	0.16 ± 0.073	130.79	<0.001
Methylmalonylcarnitine/3-Hydroxyisovalerylcarnitine (C4DC + C5OH)	0.20 ± 0.057	0.21 ± 0.058	0.21 ± 0.058	0.21 ± 0.058	0.22 ± 0.066	0.22 ± 0.060	0.22 ± 0.067	154.19	<0.001
Tiglylcarnitine (C5:1)	0.036 ± 0.017	0.036 ± 0.017	0.036 ± 0.017	0.035 ± 0.016	0.034 ± 0.016	0.033 ± 0.015	0.037 ± 0.018	56.61	<0.001
Glutarylcarnitine/3-Hydroxyhexanoylcarnitine (C5DC + C6OH)	0.18 ± 0.059	0.19 ± 0.059	0.19 ± 0.059	0.19 ± 0.058	0.18 ± 0.058	0.18 ± 0.054	0.18 ± 0.057	45.50	<0.001
Adipylcarnitine (C6DC)	0.18 ± 0.071	0.19 ± 0.073	0.19 ± 0.074	0.19 ± 0.073	0.19 ± 0.071	0.18 ± 0.067	0.20 ± 0.075	40.16	<0.001
Octenoylcarnitine (C8:1)	0.16 ± 0.060	0.17 ± 0.058	0.17 ± 0.057	0.17 ± 0.057	0.17 ± 0.057	0.17 ± 0.056	0.17 ± 0.053	15.71	<0.001
Decenoylcarnitine (C10:1)	0.093 ± 0.036	0.099 ± 0.036	0.098 ± 0.034	0.097 ± 0.034	0.097 ± 0.033	0.095 ± 0.030	1.00 ± 0.028	33.50	<0.001
Decadienoylcarnitine (C10:2)	0.030 ± 0.012	0.030 ± 0.012	0.030 ± 0.012	0.029 ± 0.015	0.029 ± 0.011	0.028 ± 0.011	0.030 ± 0.011	35.46	<0.001
Dodecenoylcarnitine (C12:1)	0.092 ± 0.050	0.11 ± 0.054	0.12 ± 0.052	0.11 ± 0.051	0.11 ± 0.051	0.12 ± 0.051	0.11 ± 0.049	134.97	<0.001
Tetradecenoylcarnitine (C14:1)	0.12 ± 0.055	0.14 ± 0.058	0.14 ± 0.055	0.13 ± 0.052	0.13 ± 0.051	0.13 ± 0.053	0.13 ± 0.053	148.37	<0.001
Tetradecadienoylcarnitine (C14:2)	0.311 ± 0.011	0.033 ± 0.011	0.032 ± 0.011	0.032 ± 0.010	0.031 ± 0.010	0.031 ± 0.0098	0.032 ± 0.0091	122.95	<0.001
3-Hydroxymyristoylcarnitine (C14OH)	0.023 ± 0.011	0.026 ± 0.011	0.026 ± 0.011	0.026 ± 0.011	0.026 ± 0.011	0.026 ± 0.011	0.026 ± 0.012	102.42	<0.001
Palmitoleoylcarnitine (C16:1)	0.14 ± 0.093	0.18 ± 0.087	0.18 ± 0.081	0.18 ± 0.080	0.18 ± 0.081	0.19 ± 0.083	0.18 ± 0.092	269.34	<0.001
3-Hydroxypalmitoleoylcarnitine (C16:1OH)	0.041 ± 0.015	0.047 ± 0.014	0.046 ± 0.014	0.047 ± 0.014	0.047 ± 0.014	0.047 ± 0.013	0.049 ± 0.013	188.56	<0.001
3-Hydroxypalmitoylcarnitine (C16OH)	0.026 ± 0.012	0.030 ± 0.012	0.030 ± 0.012	0.029 ± 0.012	0.029 ± 0.012	0.030 ± 0.012	0.029 ± 0.013	111.87	<0.001
Oleoylcarnitine (C18:1)	1.29 ± 0.51	1.43 ± 0.43	1.37 ± 0.40	1.35 ± 0.39	1.36 ± 0.40	1.40 ± 0.40	1.36 ± 0.41	111.25	<0.001
3-Hydroxyoleoylcarnitine (C18:1OH)	0.028 ± 0.011	0.031 ± 0.010	0.031 ± 0.010	0.031 ± 0.010	0.031 ± 0.010	0.031 ± 0.010	0.029 ± 0.010	70.87	<0.001
Linoleoylcarnitine (C18:2)	0.25 ± 0.11	0.21 ± 0.093	0.19 ± 0.087	0.19 ± 0.084	0.19 ± 0.083	0.18 ± 0.083	0.22 ± 0.10	534.17	<0.001
3-Hydroxystearoylcarnitine (C18OH)	0.019 ± 0.0089	0.022 ± 0.0091	0.022 ± 0.0089	0.022 ± 0.0086	0.022 ± 0.0086	0.022 ± 0.0085	0.022 ± 0.0084	138.13	<0.001
Others (μmol/L)									
Succinylacetone (SA)	0.86 ± 0.32	0.88 ± 0.32	0.87 ± 0.32	0.87 ± 0.32	0.87 ± 0.32	0.88 ± 0.31	0.85 ± 0.30	2.21	1.00

Data are expressed as mean (standard deviation). SD, standard deviation.

a *F* values derived from one-way analysis of variance (ANOVA).

b *P* values were adjusted using the Bonferroni correction for multiple comparisons.

**Table 3:** Results of two-way ANOVA evaluating the independent and interactive effects of gestational age and birth weight.

Analyte	Main effect: GA	Main effect: BW	Interaction: GA × BW
	*F* value	*F* value	*F* value (*P* value)
Amino Acids			
ALA	134.80	217.34	**30.46 (<0.001)**
ARG	375.93	12.61	**130.59 (<0.001)**
CIT	158.51	902.76	27.59 (<0.001)
GLY	207.97	216.20	85.88 (<0.001)
LEU + ILE + PRO − OH	81.42	19.99	3.965 (0.031)
MET	17.08	57.82	7.90 (<0.001)
ORN	284.45	556.68	20.34 (<0.001)
PHE	30.74	63.52	25.31 (<0.001)
PRO	322.92	1,230.57	51.42 (<0.001)
SA	2.21	0.12	1.34 (1.00)
TYR	396.08	33.93	63.95 (<0.001)
VAL	434.86	14.27	19.14 (<0.001)
Acylcarnitines			
C0	467.81	694.36	22.44 (<0.001)
C2	124.38	11.23	71.08 (<0.001)
C3	359.94	3,250.92	79.05 (<0.001)
C3DC_C4OH	131.15	11.32	66.47 (<0.001)
C4	158.05	86.25	40.03 (<0.001)
C4DC_C5OH	154.36	38.74	21.59 (<0.001)
C5	817.97	60.26	14.34 (<0.001)
C5_1	56.62	15.41	1.96 (1.00)
C5DC_C6OH	45.57	136.24	19.77 (<0.001)
C6	10.32	58.61	4.92 (0.002)
C6DC	40.17	0.77	9.80 (1.00)
C8	9.68	52.83	3.61 (0.06)
C8_1	15.71	22.62	6.11 (<0.001)
C10	94.30	202.61	46.03 (<0.001)
C10_1	33.59	268.52	16.91 (<0.001)
C10_2	35.46	0.54	1.83 (1.00)
C12	182.41	165.94	94.94 (<0.001)
C12_1	135.35	52.61	60.89 (<0.001)
C14	194.56	12.24	150.93 (<0.001)
C14_1	148.87	17.25	82.32 (<0.001)
C14_2	123.27	209.01	29.59 (<0.001)
C14OH	102.58	10.66	37.28 (<0.001)
C16	452.52	191.98	183.74(<0.001)
C16_1	271.11	95.75	146.63 (<0.001)
C16_1OH	189.47	263.69	76.14 (<0.001)
C16OH	112.11	10.93	52.46 (<0.001)
C18	262.83	20.27	149.65 (<0.001)
C18_1	111.78	5.74	117.42 (<0.001)
C18_1OH	70.99	4.95	42.31 (<0.001)
C18_2	537.27	689.34	29.03 (<0.001)
C18OH	138.51	130.60	47.10 (<0.001)

Bold values indicates statistically significant difference at *P*<0.05.

**Figure 1:**
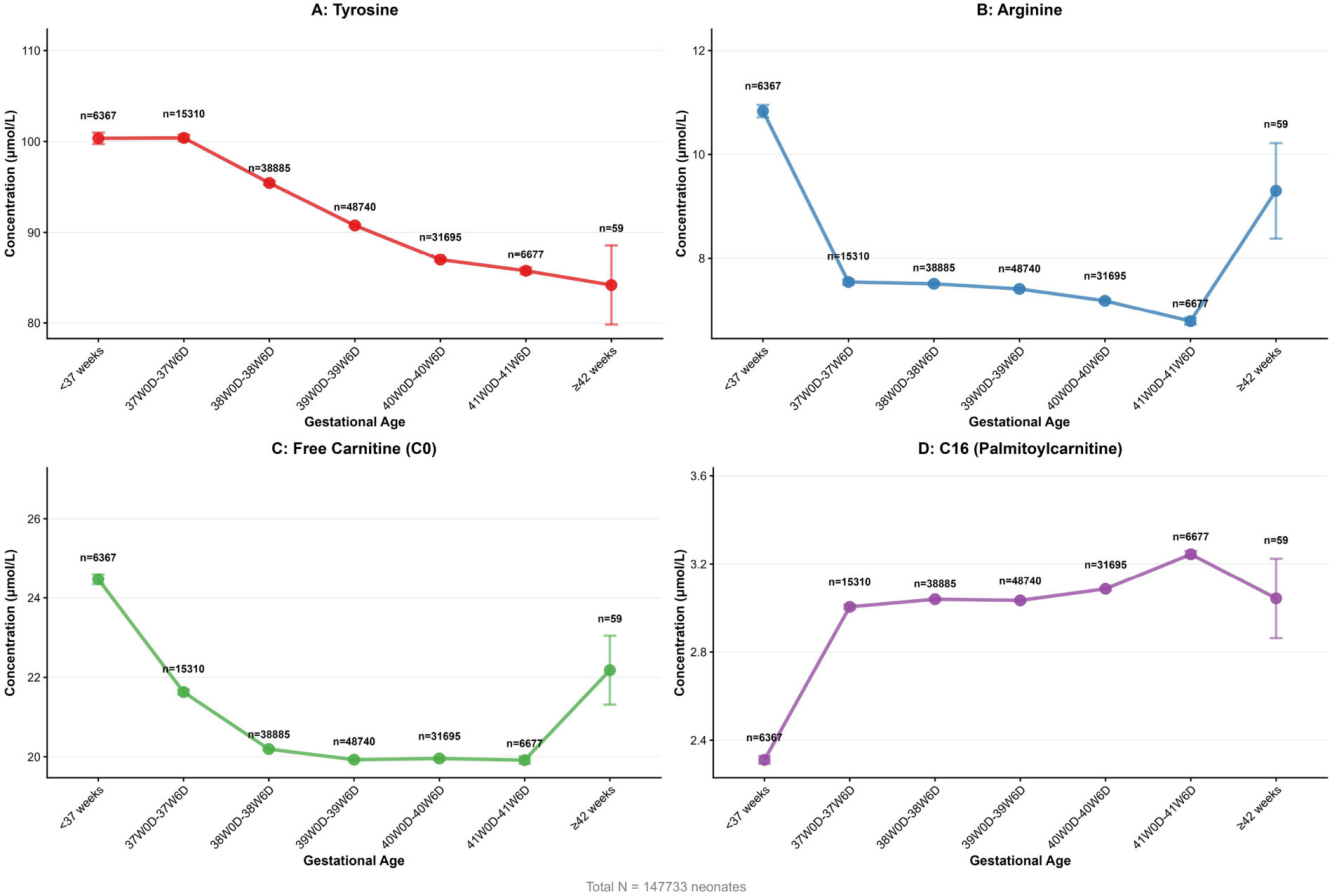
Metabolic profiles across gestational age. **(A)** Tyrosine; **(B)** Arginine; **(C)** Free Carnitine (C0); **(D)** C16 (Palmitoylcarnitine).

A clear developmental gradient was observed: concentrations of most amino acids decreased with advancing gestational age. For example, tyrosine (TYR) levels were markedly elevated in preterm infants (<37 weeks) compared with term infants, a pattern consistent with relative hepatic immaturity and reduced enzymatic clearance (Figure 1).

In contrast, fatty acid oxidation markers showed distinct maturational trajectories. Free carnitine (C0) levels declined progressively with advancing gestational age, whereas several long-chain acylcarnitines displayed gestation-dependent increases, suggesting progressive maturation of mitochondrial lipid oxidation pathways.

#### 3.2.1 Interaction effects of gestational age and birth weight

Beyond the independent main effects, two-way ANOVA revealed widespread interaction effects between gestational age and birth weight, supporting a synergistic interplay between intrauterine duration and fetal growth in shaping neonatal metabolic phenotypes (Table 3).

**Figure 2:**
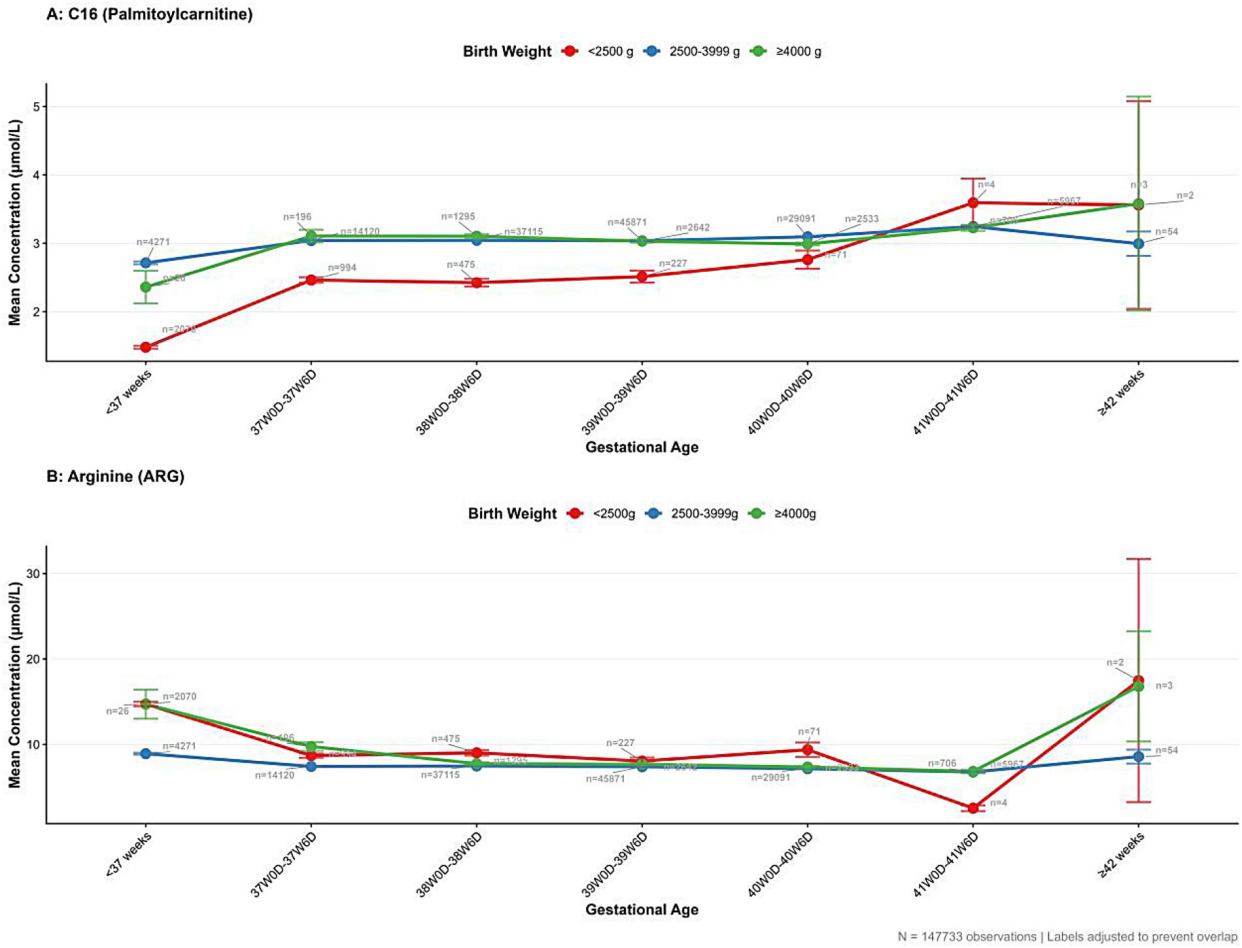
Interaction effects of gestational age and birth weight. **(A)** C16 (Palmitoylcarnitine); **(B)** Arginine (ARG).

The non-uniform impact of birth weight across gestational age groups is visualized in Figure 2. For long-chain acylcarnitines, Palmitoylcarnitine (C16) demonstrated the strongest interaction effect (F = 183.74, *P* < 0.001). While C16 concentrations generally increased with gestational maturity (as seen in the main effects), the disparity between low birth weight (<2,500 g) and normal weight infants was disproportionately amplified in the preterm cohort compared to term infants (Figure 2A). This suggests that the metabolic deficit in fatty acid oxidation markers associated with low birth weight is exacerbated by physiological prematurity.

Similarly, amino acid profiles, particularly arginine (ARG), exhibited distinct interaction patterns (F = 130.59, *P* < 0.001; Figure 2B). The elevation of arginine levels typically observed in lower-birth-weight infants was substantially more pronounced in preterm neonates, further indicating that fetal growth restriction and developmental immaturity jointly influence amino acid homeostasis.

### 3.3 Multivariate pattern recognition and metabolic clustering

**Figure 3:**
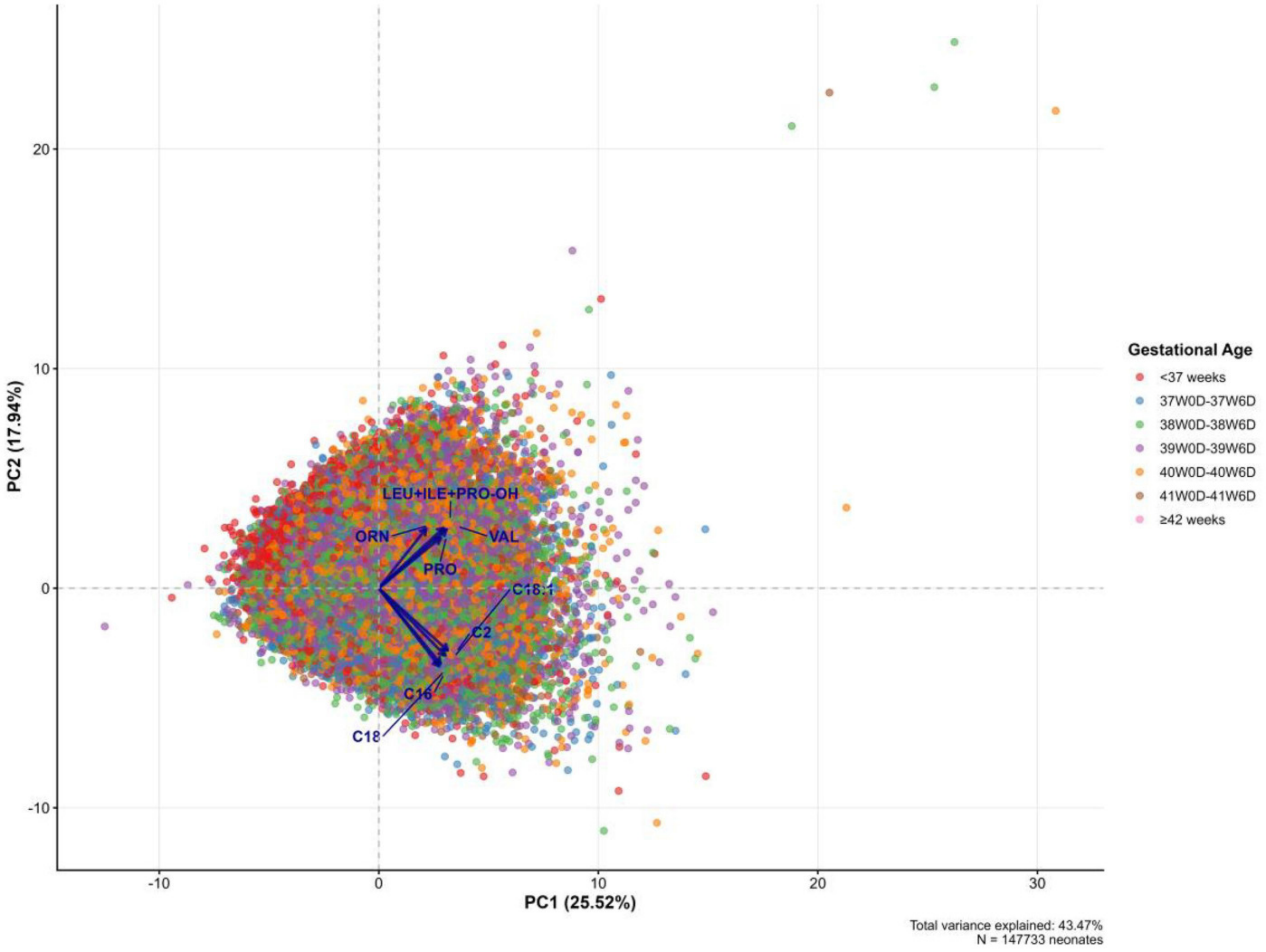
PCA biplot of metabolic profiles.

To visualize the global metabolic architecture and identify systemic patterns extending beyond individual biomarkers, principal component analysis (PCA) was performed. As illustrated in the PCA biplot (Figure 3), the first two principal components captured a biologically interpretable gradient of metabolic maturation.

The first principal component (PC1), accounting for the majority of the variation (26.28%), was heavily and positively loaded by long-chain and medium-chain acylcarnitines—specifically C14, C16, C2, and C18—serving as a proxy for fatty acid oxidation capacity. The second principal component (PC2, 19.65%) was primarily driven by branched-chain amino acids (LEU + ILE + PRO-OH, VAL) and urea cycle metabolites (ORN, PRO), reflecting protein turnover status.

The score plot revealed a distinct developmental gradient along these orthogonal axes. Preterm infants (<37 weeks) clustered predominantly in the quadrant characterized by negative PC1 scores and positive PC2 scores, whereas term infants were distributed toward regions consistent with greater metabolic maturity and more coordinated fatty acid oxidation.

**Figure 4:**
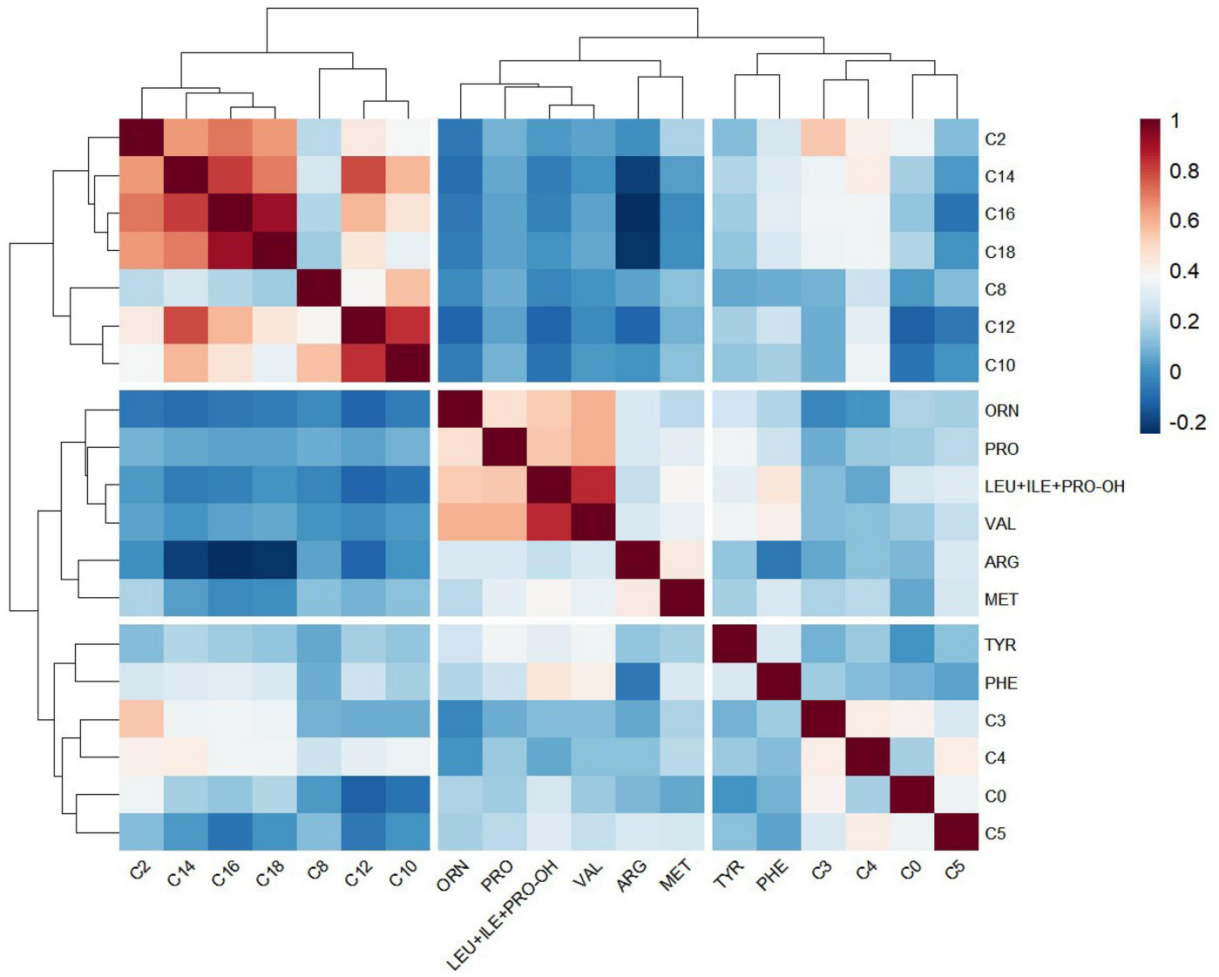
Metabolic correlation heatmap (pheatmap).

To further corroborate these inter-metabolite relationships, a correlation heatmap was generated (Figure 4). The analysis highlighted a structured modularity within the metabolome. A robust, tightly interconnected cluster was observed among acylcarnitines, particularly long-chain species, with Palmitoylcarnitine (C16) exhibiting a near-linear correlation with Stearoylcarnitine (C18) (*r* = 0.90) and Myristoylcarnitine (C14) (*r* = 0.81).

Distinct from this lipid oxidation module, amino acids formed a separate correlation block. Specifically, branched-chain amino acids showed strong internal consistency, exemplified by the high correlation between Valine and Leucine + Isoleucine (*r* = 0.84). Notably, the cross-correlation between the acylcarnitine and amino acid clusters was generally weak. This modular architecture confirms that physiological maturation manifests as coordinated shifts in specific metabolic pathways rather than isolated biomarker fluctuations.

## 4 Discussion

This large retrospective study of more than 147,000 neonates provides a comprehensive overview of metabolic maturation across the gestational spectrum. Our findings demonstrate that neonatal metabolite profiles are influenced not only by gestational age and birth weight individually, but also by their interaction, underscoring the limitations of applying uniform newborn screening thresholds across heterogeneous neonatal subgroups.

The inverse association between amino acid concentrations and gestational age is biologically plausible and consistent with the physiological immaturity of hepatic and renal systems in premature infants (14). The marked elevation of tyrosine in preterm neonates may reflect delayed maturation of tyrosine catabolic pathways, whereas higher arginine concentrations may be related to developmental differences in nitrogen handling and enteral nutritional exposure (15, 16).

A notable finding of this study is the fatty acid oxidation profile observed in preterm infants: relatively elevated free carnitine (C0) accompanied by reduced long-chain acylcarnitines such as C16. This pattern may reflect incomplete mitochondrial transport and *β*-oxidation capacity, together with altered substrate availability in early life (17–19).

Perhaps the most clinically meaningful result is the significant interaction between gestational age and birth weight. Our data indicate that the metabolic effect of birth weight is context dependent rather than uniform (20, 21). In practical terms, a low birth weight in a preterm infant does not carry the same biochemical implications as a similar birth weight in a term infant, reinforcing the need for stratified and clinically interpretable screening cutoffs.

The systemic metabolic shifts identified by PCA and correlation analyses further challenge the traditional “one-size-fits-all” approach to newborn screening (22). The modular behavior of acylcarnitines and amino acids suggests that metabolic immaturity affects coordinated biochemical pathways rather than isolated markers alone.

The strengths of this study include its large sample size and the explicit statistical evaluation of interaction effects, which have often been overlooked in prior single-factor analyses. Several limitations should also be acknowledged. First, because the study was retrospective, detailed individual-level data on parenteral nutrition, specific enteral supplements, and concurrent clinical conditions were not available for all infants. Second, the cohort was derived from a single provincial screening center, which may limit generalizability to other populations. Third, although the findings support refinement of screening interpretation, disease-confirmed outcome analyses were beyond the scope of the present work.

## 5 Conclusion

In conclusion, this study delineates the metabolic landscape of the neonatal period and shows that metabolic maturity is jointly shaped by gestational age and birth weight. The observed subgroup-specific patterns provide supportive evidence for more individualized and developmentally informed interpretation of newborn screening results.

## Data Availability

The original contributions presented in the study are included in the article/Supplementary Material, further inquiries can be directed to the corresponding author.
